# Conformational Fingerprints Underlying Thermal Modulation of PAC/TMEM206 Gating

**DOI:** 10.3390/ijms27114784

**Published:** 2026-05-26

**Authors:** Rachel Reyes-Lizana, German Fernández, Scarleth Duran-Morales, Guillermo Carrasco-Faus, Yorley Duarte, Valeria Marquez-Miranda, Ignacio Díaz-Franulic

**Affiliations:** Center for Bioinformatics and Integrative Biology, Universidad Andres Bello, Santiago 8370146, Chile

**Keywords:** PAC/TMEM206, temperature modulation, intersubunit coupling

## Abstract

The proton-activated chloride channel PAC/TMEM206 is broadly expressed in mammalian tissues and contributes to acid-induced cell injury in pathological settings such as ischemia, inflammation, and tumor acidosis. In addition to its established proton sensitivity, PAC is strongly modulated by temperature: heating potentiates proton-evoked currents and shifts activation toward less acidic pH yet does not open the channel at neutral pH. How proton and thermal inputs are structurally integrated remains unclear. Here, we combined site-specific incorporation of the environmentally sensitive fluorescent amino acid ANAP with automated patch-clamp electrophysiology and molecular dynamics simulations to identify structural elements underlying PAC thermal modulation. ANAP reporters introduced across the extracellular domain and vestibule–pore coupling region revealed residue-specific thermal- and proton-dependent spectral shifts, showing that heating and acidification remodel overlapping but non-identical local environments. The strongest ANAP-reported temperature-dependent changes were observed at R93, Y111, F196, R237, and F282, whereas proton-dependent changes prominently involved R93, Y111, H130, and R237. Several temperature-sensitive ANAP reporters mapped to an intersubunit region also highlighted by dynamic correlation analysis. Together, our results identify structural correlates of PAC thermal modulation consistent with a model in which protonation creates an activation-permissive landscape while heating reweights coupling across an intersubunit scaffold; this model generates testable predictions that should be addressed by targeted mutagenesis and thermodynamic characterization.

## 1. Introduction

Acidosis is a fundamental challenge faced by cells and tissues under both physiological and pathological conditions. Acid-base disturbances occur across multiple levels of biological organization. At the systemic level, extracellular acidification may result from respiratory failure (hypercapnia), metabolic disorders such as diabetic ketoacidosis, lactic acidosis in sepsis, or renal tubular acidosis. In these scenarios, systemic extracellular pH may drop significantly, compromising multiple organ systems [1]. At the tissue level, localized extracellular acidosis is a hallmark of many pathological environments. Ischemic tissues rapidly acidify due to anaerobic glycolysis and CO_2_ accumulation [2]. Inflammatory sites also exhibit acidification secondary to leukocyte activation and local hypoxia [3]. Perhaps most strikingly, solid tumors develop a highly acidic microenvironment because of the Warburg effect [4], sustaining an inverted pH gradient that promotes cell proliferation, extracellular matrix remodeling, metastatic potential [5], immune evasion, and resistance to chemotherapy [6]. At the cellular level, cells rely on a vast repertoire of molecular proton sensors that detect extracellular acidification and initiate appropriate responses to maintain homeostasis or trigger cell fate decisions. These pH sensors are particularly well developed in excitable cells such as neurons, where rapid pH fluctuations can occur during synaptic transmission, hypoxia, and ischemic injury. In neurons, acid-sensing ion channels (ASICs) serve as key proton detectors [7]; they are voltage-insensitive, sodium-permeable channels that open upon extracellular acidification, contributing to pain perception, mechanotransduction, synaptic plasticity, and ischemic neurotoxicity [8]. Other neuronal proton sensors include voltage-gated proton channels (Hv1), which extrude protons during oxidative bursts [9], and proton-sensitive GPCRs that modulate signaling pathways [10]. Unlike excitable cells, which rely on ASICs and voltage-gated proton channels to rapidly sense and respond to extracellular pH fluctuations, non-excitable cells must employ distinct molecular strategies to regulate homeostasis under acidic stress. While a variety of secondary transporters such as Na^+^/H^+^ exchangers, Cl^−^/HCO_3_^−^ exchangers, and proton-coupled monocarboxylate transporters indirectly contribute to pH regulation [11,12] very few ion channels are known to sense extracellular protons in these cellular contexts directly. One of the earliest descriptions of a proton-activated conductance in non-excitable cells was the acid-sensitive outwardly rectifying chloride current (ASOR), a chloride current that is robustly activated by extracellular acidification and has been observed across multiple mammalian cell types [13], including fibroblasts, epithelial cells [14], and cancer-derived cell lines [15]. Despite extensive biophysical characterization, the molecular identity of ASOR remained elusive for nearly two decades. The recent identification of PAC (TMEM206) as the molecular correlate of ASOR represents a breakthrough in the field of proton sensing in non-excitable cells [16,17]. PAC is broadly expressed across epithelial tissues, immune cells, neurons, and tumor cells, and is highly conserved across vertebrate species. Functional studies have shown that PAC mediates chloride influx in response to extracellular acidification, contributing to cell volume dysregulation, acid-induced cell death, and tissue injury in various conditions, including ischemia, stroke, and inflammation [18]. PAC activation has also been implicated in tumor acidosis, where its activity may influence cancer cell survival, invasiveness, and resistance to therapy within the acidic tumor microenvironment [19,20,21]. In addition to its well-established proton sensitivity, PAC exhibits a pronounced temperature dependence: elevated temperatures shift its pH activation curve toward less acidic values and markedly enhance current amplitude [22,23]. Importantly, temperature alone does not activate PAC at neutral pH, indicating that heat modulates gating only within the proton-bound conformational ensemble. This conditional thermal responsiveness distinguishes PAC from channels that can be directly thermoactivated [24,25] and suggests that temperature reshapes the energetic landscape of proton-driven activation rather than acting as an independent stimulus. Multiple molecular frameworks have been proposed to explain temperature gating in ion channels, including models based on changes in heat capacity associated with large-scale buried-to-exposed transitions of hydrophobic residues, as well as models invoking distributed allosteric reorganization of interaction networks [26,27,28,29]. Whether PAC temperature sensitivity arises from global solvent exposure changes, from localized structural rearrangements, or from modulation of interdomain coupling remains unknown. Using ANAP fluorescence mapping together with molecular dynamics simulations, we identify several temperature-responsive elements in PAC, suggesting that heating drives a spatially organized remodeling of local chemical environments during proton gating. These findings contribute to setting a mechanistic framework for understanding how temperature modulates PAC function without acting as an independent gating stimulus.

## 2. Results

### 2.1. Temperature and pH-Dependent Displacements of ANAP Emission Spectrum Suggest Partial Overlapping Between Gating Modalities of PAC

**Figure 1:**
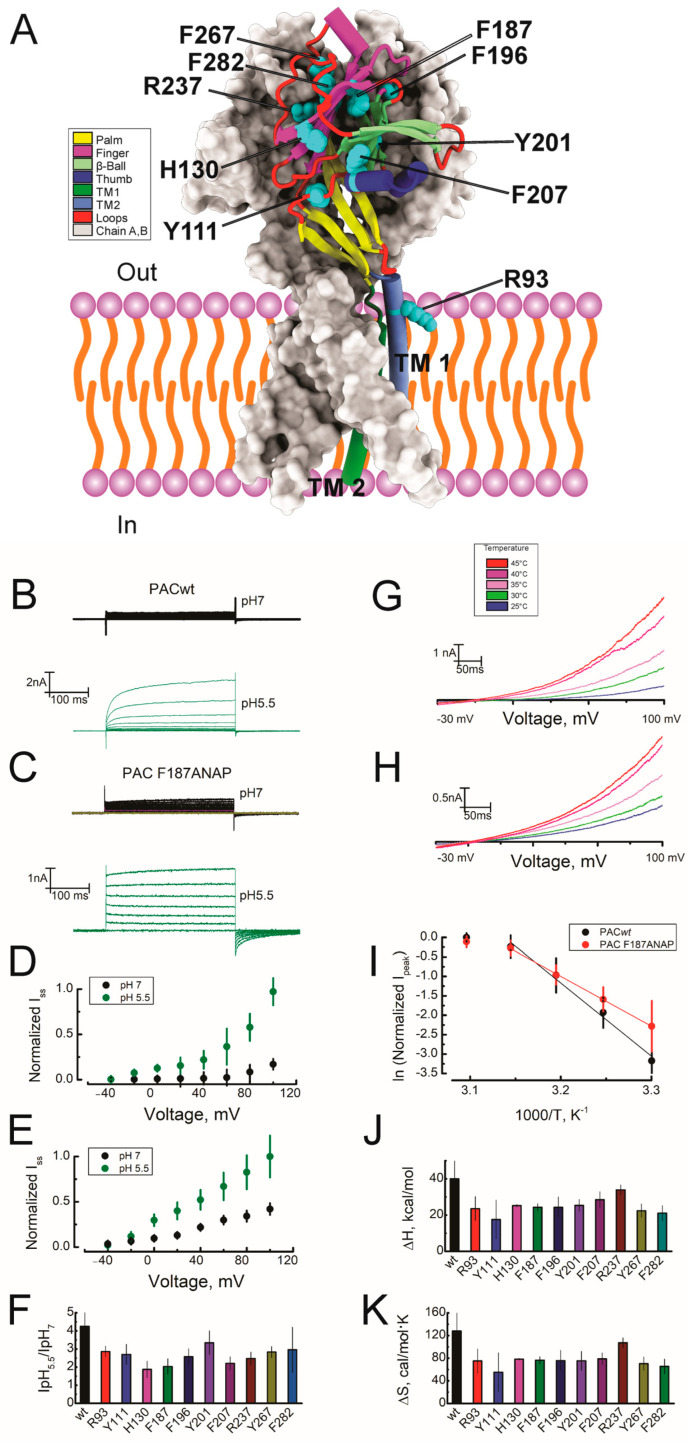
(**A**) Structural representation of human PAC showing the location of the ANAP-labeled residues analyzed in this study. The protein is shown as a surface-rendered trimer, with one subunit highlighted in cartoon representation. Structural elements are color-coded as indicated: palm, finger, β-ball, thumb, TM1, TM2, and loops; the other subunits are shown in gray surface representation. The approximate membrane boundaries are indicated, with extracellular (Out) and intracellular (In) sides labeled. (**B**) Representative whole-cell currents recorded from the same HEK293 *tmem206*^−/−^ cell expressing PAC WT at pH 7.0 (black) and pH 5.5 (green), elicited by voltage steps from −40 to +100 mV in 20-mV increments. (**C**) Representative whole-cell currents recorded from the same HEK293 *tmem206*^−/−^ cell expressing PAC F187ANAP at pH 7.0 (black) and pH 5.5 (green), using the same voltage protocol. (**D**) Corresponding normalized steady-state current–voltage relationship for PAC WT at pH 7.0 and pH 5.5. Data are shown as mean ± SEM. (**E**) Corresponding normalized steady-state current–voltage relationship for PAC F187ANAP at pH 7.0 and pH 5.5. Data are shown as mean ± SEM. (**F**) Summary of steady-state current amplitude at +100 mV, expressed as the ratio IpH5.5/IpH7.0, for PAC WT and the indicated ANAP-substituted variants. Data are shown as mean of 4–6 cells ± SEM. (**G**,**H**) Representative traces of current evoked by voltage ramps from −30 to +100 mV at temperatures ranging from 25 °C to 45 °C for PAC WT (**G**) and PAC F187ANAP (**H**) under acidic conditions (pH 5.5). (**I**) van’t Hoff plot derived from current activation measurements for PAC WT (black) and PAC F187ANAP (red). Lines represent linear fits used to estimate the apparent thermodynamic parameters of activation. (**J**,**K**) Summary of apparent activation enthalpy (ΔH, (**J**)) and entropy (ΔS, (**K**)) obtained from van’t Hoff analysis for PAC WT and the indicated ANAP-containing constructs.

Temperature-dependent changes in solvent exposure of hydrophobic amino acids have been invoked to explain the steep temperature dependence of thermoTRP channels by the hat capacity hypothesis [26,27,30]. To test whether thermal modulation of PAC is accompanied by position-specific buried/exposed transitions [31], we incorporated the polarity-sensitive unnatural amino acid ANAP at 10 sites across the extracellular domain of TMEM206 (R93, Y111, H130, F187, F196, Y201, F207, R237, F267 and F282) chosen to sample distinct structural elements of the extracellular domain and vestibule–pore coupling region. Figure 1A displays the cryo-EM structure of PAC in the resting conformation (PDB: 7SQG [32]), highlighting the 10 positions targeted for ANAP substitution. The selected residues map to spatially separated elements spanning the ECD scaffold and the pore-adjacent vestibular region, providing a distributed structural readout of local environment changes during temperature modulation. Because unnatural amino acid incorporation can perturb folding, trafficking, or gating [33], we first verified the functional integrity of each ANAP-containing construct using automated whole-cell patch-clamp electrophysiology. Figure 1B shows ionic currents evoked by voltage steps from −40 to 100 mV in patch-clamp experiments performed on HEK293 *tmem206*^−/−^ cells expressing PAC WT channels at pH 7.0 (black) and pH 5.5 (green). Figure 1C shows recordings obtained using the same protocol for PAC-F187ANAP channels, with identical color code. The lower panels quantify these experimental datasets. Figure 1D,E show the normalized steady-state current (I_ss_)-voltage relations for PAC WT and PAC F187ANAP channels, respectively, measured at pH 7.0 (black) and pH 5.5 (green). Figure 1F summarizes the I_pH 5.5_/I_pH 7_ ratio for WT and the indicated ANAP-substituted PAC variants, providing a comparative estimate of proton-dependent current enhancement. Figure 1G,H show the membrane currents evoked by a 300 ms voltage ramp from −30 to 100 mV for WT and PAC F187ANAP, respectively, at temperatures ranging from 25 to 45 °C. After the whole cell configuration is reached and I–V curve is performed at pH 7 and 5.5, a small volume of pre-heated solution is injected into each of the Patchliner chip wells while the ramp protocol is continuously executed, producing a transient increase in the current. Starting from basal temperature of 20 °C, pre-heated solution pulses at 25 (blue), 30 (green), 35 (pink), 40 (purple) and 45 (red) °C are delivered. Figure 1I presents the van’t Hoff analysis where the natural logarithm of normalized I_peak_ is plotted against 1000/T for PAC WT and PAC F187ANAP. Solid lines represent the linear fit over the temperature range where the maximal slope is achieved. Figure 1J,K summarize the thermodynamic parameters extracted from these analyses, namely the apparent enthalpic (ΔH) and entropic (ΔS) contributions for WT and the indicated PAC variants. Notably, all ANAP-substituted variants retained temperature-sensitive gating to some extent, with apparent ΔH values ranging from ~17 to ~38 kcal/mol, broadly consistent with the temperature dependence reported for PAC in native cellular contexts [22,23]. The same protocol, combining acidic conditions with temperature steps, was performed in untransfected cells and showed no significant increase in membrane currents (Figure S1). The observation that ANAP substitutions at distinct structural positions preserve qualitatively similar thermodynamic signatures suggests that no single residue dominates the thermal response and instead points toward a distributed enthalpic contribution across the extracellular domain. Taken together, these results establish that the ANAP-labeled PAC variants are functionally competent probes suitable for site-specific spectroscopic interrogation of conformational changes driven by temperature and protonation.

**Figure 2:**
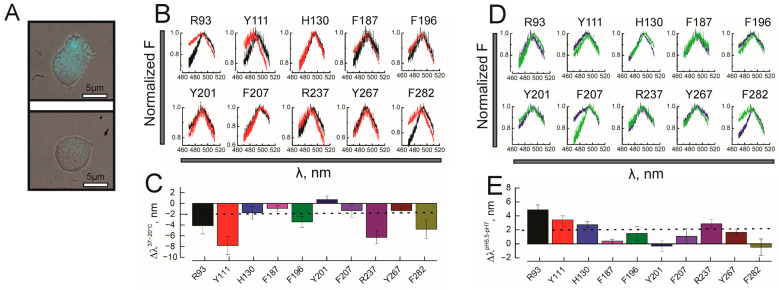
Site-specific ANAP fluorescence reveals distinct temperature- and proton-dependent changes in the local environment of PAC residues. (**A**) Representative epifluorescence image of cells expressing PAC–ANAP constructs (up) and non-transfected cells (bottom). (**B**) Normalized ANAP emission spectra recorded at 20 °C (black) and 37 °C (red) under acidic conditions for the indicated PAC positions. Spectra were normalized to facilitate comparison of temperature-dependent shifts in emission maxima. (**C**) Summary of the temperature-dependent spectral shift for each reporter position, expressed as Δλ 37–20 °C. Negative values indicate blue shifts upon heating, consistent with movement to a less polar and/or more conformationally restricted local environment. Data are shown as mean ± SEM. (**D**) Normalized ANAP emission spectra recorded at pH 7.0 (blue) and pH 5.5 (green) at 20 °C for the indicated positions. Spectra were normalized to compare pH-dependent changes in emission maxima. (**E**) Summary of the proton-dependent spectral shift for each reporter position, expressed as Δλ pH 5.5–pH 7. Positive values indicate red shifts under acidic conditions, consistent with a more polar local environment. Data are shown as mean of 7–15 cells ± SEM. Dashed lines indicate threshold values used to highlight residues with the largest spectral changes. Nonparametric bootstrap permutation testing with Benjamini–Hochberg FDR correction across all 10 positions identified R93, Y111, R237, F282 (q < 0.01) and F196 (q < 0.05) as statistically significant, consistent with the |Δλ| ≥ 2 nm threshold.

We next recorded ANAP emission spectra from PAC–ANAP constructs. As an initial control for specificity of ANAP-associated fluorescence, Figure 2A shows representative epifluorescence images of a transfected cell expressing PAC–F187ANAP (upper panel). Non-transfected cell subjected to the same ANAP incubation and imaging conditions (lower panel), which displayed only residual ANAP fluorescence. This confirms that the measured emission signal reflects channel-associated ANAP incorporation rather than nonspecific uptake of the fluorophore. We first examined temperature-dependent spectral changes under acidic, activatable conditions by recording emission spectra at 20 °C (black traces) and 37 °C (red traces) at pH 5.5 (Figure 2B). Figure 2C shows the average spectral displacement upon heating (∆λ^37–20°C^) for each PAC variant. In most cases, raising temperature shifted ANAP emission toward shorter wavelengths. While such blue shifts are commonly interpreted as reflecting reduced local polarity or hydration of the fluorophore environment [31], they may also arise from rotameric rearrangements of the ANAP side chain that reposition the probe closer to neighboring residues, thereby altering the local dielectric constant, independently of changes in solvent exposure. We cannot formally exclude this possibility, and therefore interpret the observed spectral shifts as qualitative reporters of local environmental changes rather than as unambiguous indicators of altered hydration per se [31]. Using an amplitude threshold of 2 nm to highlight significant changes [34], we observed temperature-dependent spectral shifts when ANAP was introduced at R93, Y111, F196, R237, and F282. Nonparametric bootstrap permutation testing identified these residues’ spectral shifts as statistically significant with the current population size (see Section 4). Thus, our results suggest that heating does not induce a uniform global response, but rather residue-specific changes in the ANAP environment within the acidic state, consistent with local remodeling. It is worth considering that all ANAP substitutions dampened to some extent both temperature and pH-driven channel gating, so the magnitude of spectral displacements must be considered just as a qualitative proxy of conformational changes triggered by these stimuli.

To determine whether these thermal rearrangements coincide with structural transitions driven by extracellular protons, we next compared spectra recorded at 20 °C at pH 7.2 (blue traces) and those obtained at pH 5.5 (green traces) (Figure 2D) and average spectral displacement upon acidification (∆λ^pH5.5–pH7^) are summarized in Figure 2E. Acidification alone also altered ANAP emission, but the pattern was not identical to that observed for temperature. Here, the largest pH-dependent shifts were detected at R93, Y111, H130, F207, and R237, but all of them were toward longer wavelengths, suggesting the probe becomes exposed to a more polar environment upon extracellular milieu acidification. Comparing the sets of residues with significant spectral shifts under each condition revealed a partial but meaningful overlap between temperature- and proton-dependent local rearrangements—arguably the most important observation of this section. Positions such as R93, Y111, and R237 responded to both stimuli, whereas F196, F282, and H130 showed preferential sensitivity to either heat or acidification. These results indicate that temperature and protons converge on a shared structural scaffold while each stimulus additionally engages distinct elements, consistent with a model in which protonation establishes the permissive conformational landscape while heating selectively reweights interactions within it.

### 2.2. Molecular Dynamics Simulations Reveal Temperature-Dependent Reorganization of the β12–Finger–Pore Coupling Axis in PAC

**Figure 3:**
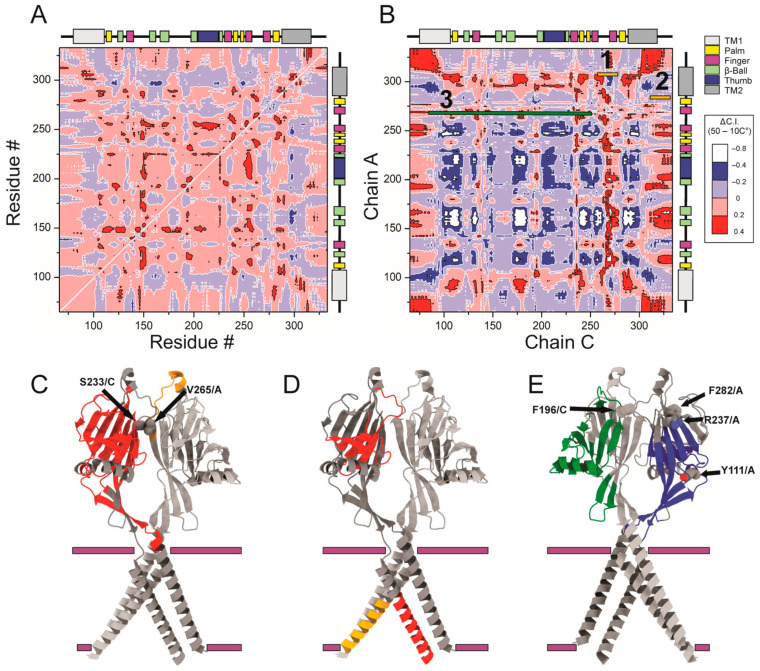
Temperature-dependent remodeling of correlated motions in PAC reveals intra- and intersubunit coupling pathways. (**A**) Differential intrasubunit cross-correlation map showing temperature-dependent changes in residue-residue correlated motions within PAC subunits. Domain organization is shown above and to the right of the map. (**B**) Differential intersubunit cross-correlation map showing temperature-dependent changes in correlated motions between neighboring PAC subunits A and C. Numbered regions indicate clusters of intersubunit correlation changes selected for structural mapping in panels (**C**–**E**). (**C**) Structural mapping of intersubunit correlated cluster 1 in panel (**B**), with subunit A residues 264–276 shown in orange and subunit C residues 95–188, 198–209, 229–248, 257–266, and 278–291 shown in red. Black arrows indicate the residues located closest to the interface between the two subunits, V265 (chain A) and S233 (chain C). (**D**) Structural mapping of intersubunit correlated cluster 2 in panel (**B**), with subunit C residues 315–332 shown in orange and subunit A residues 114–121, 150–171, 191–206, and 312–333 shown in red. (**E**) Structural mapping of the large intersubunit anticorrelated cluster labeled 3 in panel (**B**), with subunit A residues 98–119, 168–185, 201–213, 228–232, 238–251, 254–262, and 283–300 shown in blue, and subunit C residues 113–117, 146–172, 188–R233, and 245–257 shown in green. Black arrows indicate residues showing significant temperature-dependent spectral shifts, Y111, R237 and F282 (chain A) and F196 (chain C). Horizontal bars indicate the boundaries of the lipid bilayer.

The temperature and pH-dependent ANAP spectral changes indicate that heating and acidification affect overlapping, but non-identical, structural elements. This observation prompted us to ask whether temperature might also alter the conformational dynamics of PAC outside the protonated ensemble. We therefore examined the effect of temperature on the resting-state structure using molecular dynamics simulations. at 10 °C and 50 °C. In both conditions, the trimer remained globally stable throughout the simulations, without evidence of unfolding, although structural deviations were modestly larger at 50 °C. Root mean square fluctuations analysis (RMSF) showed that the effect of heating was not uniformly distributed across the channel, but instead localized to discrete regions, including the N-terminal portion of TM1, the Palm domain, the extracellular core, and the C-terminal region of TM2 (Figure S2A). Consistent with this, SASA analysis revealed that temperature-dependent changes were spatially restricted rather than global, involving selected portions of the upper extracellular region, the central β-sheet scaffold, subunit interfaces, and the lower pore-coupling region (Figure S2B). Notably, the magnitude of the spectral shift did not correlate with temperature-dependent changes in SASA from molecular simulations, suggesting that the signal reflects changes in the local electrostatic environment driven by side-chain reorientation rather than altered solvent exposure (Figure S2C). We next examined temperature-dependent changes in dynamic coupling. The differential intrasubunit cross-correlation map showed relatively modest changes overall (Figure 3A), indicating that heating produced limited reorganization of concerted motions within individual subunits. In contrast, the intersubunit differential cross-correlation map between chains A and C revealed broader and more spatially organized changes between neighboring subunits (Figure 3B). These temperature-dependent changes were distributed across multiple extracellular and pore-proximal regions, indicating that the strongest effect of heating on dynamic coupling was observed at the interface between adjacent monomers.

To visualize the main intersubunit regions identified in Figure 3B, the corresponding segments were mapped onto the PAC structure. Cluster 1 (Figure 3C) comprises extensive regions of chain A, including the palm, β-ball, finger, and upper TM2 domains (red), together with a discrete segment of the finger domain from chain C (orange). V265 and S233 from chains A and C, respectively, form the closest intersubunit contact at this interface (black arrows). Notably, Y267ANAP—located only two residues away from this contact point—did not display a significant temperature-dependent spectral shift, suggesting that this interfacial region constitutes a relatively static structural anchor that physically connects neighboring subunits without undergoing substantial local environmental remodeling upon heating. This is consistent with a model in which dynamic coupling across the subunit interface is transmitted through this contact point. Cluster 2 (Figure 3D) comprises a region in chain A spanning the β-ball and finger domains and extending into a substantial portion of the C-terminal lower TM2 segment (red), with a corresponding region in chain C shown in orange. In contrast, cluster 3 (Figure 3E), represents a large anticorrelated region involving the upper TM1, β-ball, thumb, and finger domains of chain A (green) and the upper TM1, β-ball, thumb, and palm domains of chain C (blue). Strikingly, ANAP positions Y11, R237 and F282 from chain A, and F196 from chain C—all of which showed significant temperature-dependent spectral shifts—map within these anticorrelated domains, suggesting they report on thermally sensitive intersubunit conformational coupling. Together, these analyses show that heating produces detectable but limited changes in intrasubunit dynamic coupling in the resting-state model, whereas the most pronounced reorganization occurs at intersubunit interfaces, involving both correlated and anticorrelated motions across neighboring extracellular regions and pore-proximal segments. These findings suggest that thermal modulation of PAC engages both interface-centered reorganization and longer-range allosteric communication, consistent with a distributed rather than localized mechanism of temperature sensing.

## 3. Discussion

PAC integrates extracellular protons and temperature to regulate chloride conduction, yet how these two inputs are integrated structurally remains incompletely understood. Our findings, combining ANAP spectroscopy and molecular simulations, suggest that temperature acts not as an independent trigger, but as a modulator of a proton-enabled allosteric scaffold. This aligns with functional data showing that while PAC activation is strictly pH-dependent, as heating shifts the equilibrium toward activation at less acidic pH [32,35]. The ANAP measurements provide direct experimental evidence that heating under acidic conditions remodels PAC in a site-specific manner. Temperature-dependent spectral shifts were detected at only a subset of the positions examined, arguing against a non-specific thermal effect on the fluorophore environment (Figure 2). Although the mechanistic origin of these spectral changes cannot be unambiguously assigned—as blue shifts may reflect reduced solvent exposure, tighter local packing, or rotameric rearrangements of the ANAP side chain that alter the local dielectric environment—their residue-specificity strongly suggests that they report genuine, position-dependent conformational changes rather than non-specific fluorophore behavior (Figure 2). The affected positions map to structurally meaningful regions, including the distal Finger, the β-ball, and the ECD–TMD interface, all of which have previously been implicated in PAC gating and pH-dependent conformational coupling [32,36,37]. Specifically, structural comparisons show that the Finger and Thumb domains undergo significant outward displacements (>10 Å) upon acidification, acting as the primary sensors for extracellular signals. Our observation of temperature-dependent shifts in these same clusters makes it plausible that heat may prime these domains for their large-scale functional movements [36,38]. An important feature of the ANAP dataset is that the thermal and proton-dependent spectral patterns only partially overlapped. Positions such as R93, Y111, and R237 responded to both stimuli, whereas others showed preferential sensitivity to either heat or acidification. This indicates that heating does not merely reproduce the conformational changes elicited by extracellular protons. Rather, the two inputs remodel overlapping but non-identical local environments within the same broader gating architecture. This point is central because it argues against a simple linear model in which thermal potentiation is just an extension of proton binding. Instead, the data supports a view in which extracellular protonation establishes a gating-competent scaffold, while heating selectively reweighs interactions within that scaffold. This interpretation is further supported when the ANAP results are considered together with the dynamic correlation analysis. Although the simulations were performed on the resting-state structure (PDB: 7SQG) and therefore do not directly reproduce the protonated, activation-competent ensemble, the dynamic fingerprints identified in this state are nonetheless informative for understanding the activation process under acidic conditions. Specifically, the intersubunit coupling pathways that are most sensitive to temperature in the resting state overlap with structural elements known to undergo large-scale rearrangements upon acidification, including the Finger, Thumb, and β-ball domains [36,38]. This suggests that the resting-state scaffold already encodes the dynamic predispositions that are selectively amplified by protonation. In other words, the regions identified as thermally labile in the resting state may correspond to the same elements that become conformationally committed during acid-induced gating, consistent with a model in which temperature biases the pre-existing dynamic landscape of PAC toward an activation-permissive configuration rather than inducing a qualitatively new structural transition. In particular, the differential cross-correlation maps revealed that the clearest temperature-dependent reorganization occurred not within isolated subunits, but across intersubunit interfaces. This is especially relevant because several of the experimentally identified ANAP reporters with the strongest thermal responses, including F196, R237, and F282, are embedded in a network of intersubunit couplings whose dynamic weighting is altered by temperature (Figure 3E). This interpretation aligns well with current mechanistic models of PAC gating. Previous studies have identified several residues and structural elements that contribute to proton sensing and pH-dependent activation, including H98, D269, and the β12/joint region, as well as the critical interaction between H130/H131 and the acidic residue D297 in the neighboring subunit, which stabilizes the protonated ensemble [36]. By showing that temperature reorganizes these intersubunit pathways, our data suggest that thermal energy modulates the stability of these proton-sensitive pairs even before total acidification [36,37]. Our results do not argue for a distinct temperature-sensing domain separate from this machinery. Instead, they suggest that temperature may bias structural elements embedded within the broader proton-coupled gating architecture [39]. In this framework, we propose that extracellular acidification establishes the permissive conformational landscape required for activation, whereas heating reshapes the weighting of local environments and dynamic couplings within that broader scaffold. An additional implication of our data is that PAC temperature sensitivity may emerge from structural preconditioning rather than direct thermal opening. Since PAC does not activate at neutral pH in response to temperature alone [23], any thermally induced remodeling of the resting scaffold must remain functionally silent until protonation occurs. However, such remodeling could still alter the conformational predisposition of the channel, making subsequent proton-dependent activation more favorable. This possibility is consistent with the experimental observation that heating and protonation affect overlapping, but non-identical, regions of the channel, and with the simulation results showing that temperature reorganizes selected dynamic features of the resting structure without inducing global destabilization. Extracellular acidification may create a gating-competent condition, while heating reshapes local environments and intersubunit communication pathways in ways that may facilitate activation once protonation occurs. In this view, temperature acts as a modulatory input that biases the structural landscape of PAC, rather than as an independent trigger of channel opening.

## 4. Materials and Methods

### 4.1. Molecular Biology

Wild-type human PAC (hPAC/TMEM206) cloned into a pcDNA3.1 expression vector was used as the template for all constructs. To enable 3-[(6-acetyl-2-naphthyl) amino]-L-alanine (L-ANAP) incorporation by amber suppression, TAG stop codons were introduced at selected positions by site-directed mutagenesis (Synbio Technologies, Monmouth Junction, NJ, USA). The pIRES2-hPAC plasmid was a generous gift from Zhaozhu Qiu (Addgene plasmid #129488) [17]. All constructs were verified by double-stranded DNA sequencing.

### 4.2. Cell Culture, Transfection, and UAA Incorporation

HEK293 *tmem206*^−/−^ cells were a kind gift of Dr. Thomas Jentsch (Leibniz-Forschungsinstitut für Molekulare Pharmakologie (FMP), Berlin, Germany). Cell cultures were maintained in DMEM-F12 supplemented with 10% fetal bovine serum at 37 °C in a humidified atmosphere containing 5% CO_2_. Cells were transfected with Xfect (Takara Bio, Inc., San Jose, CA, USA) using plasmids encoding TMEM206 constructs together with vectors encoding the orthogonal ANAP tRNA/aminoacyl-tRNA synthetase pair. The pANAP plasmid was a gift from Peter Schultz [40] (Addgene plasmid #48696; RRID: Addgene_48696). For ANAP-based spectroscopy experiments, TMEM206 and pANAP were co-transfected at a mass ratio of 3:1. Five hours after transfection, the medium was replaced with DMEM-F12 containing ANAP [50 µM], prepared from an ethanol stock solution and sterilized through a 0.22 μm PVDF filter. Cells were incubated in ANAP-containing medium for 48 h and subsequently maintained in ANAP-free medium for 24 h before experiments.

### 4.3. Automated Patch Clamp Electrophysiology

Automated electrophysiological recordings were performed using a Patchliner system (Nanion Technologies GmbH, Munich, Germany). Cells were freshly dissociated and maintained in suspension at 12 °C before recording. Experiments were initiated at a controlled temperature of 20 °C using medium-resistance NPC-16 chips (1.8–3 MΩ). Only recordings meeting predefined quality criteria were included in the analysis (seal resistance > 200 MΩ and series resistance < 10 MΩ). The standard extracellular solution at pH 7.4 contained (in mM): 145 NaCl, 5 KCl, 1 MgCl_2_, 2 CaCl_2_, 10 glucose, and 10 HEPES, adjusted to pH 7.4 with NaOH (320 mOsm/kg). The acidic solution (pH 5.5) had the same composition except that HEPES was replaced with 5 mM sodium citrate, and pH was adjusted with citric acid. The intracellular solution contained (in mM): 50 CsCl, 10 NaCl, 60 CsF, 20 EGTA, and 10 HEPES, adjusted to pH 7.2 (285 mOsm). Cs^+^ and F^−^ were used to suppress endogenous K^+^ currents and improve recording stability. A seal enhancer solution containing (in mM) 80 NaCl, 3 KCl, 10 MgCl_2_, 35 CaCl_2_, and 10 HEPES (pH 7.4) was transiently applied to facilitate seal formation. To monitor PAC activity and current rectification, currents were elicited using a continuous voltage-ramp protocol from −30 to +100 mV over 300 ms, applied from a holding potential of −30 mV at 5 Hz. This protocol enabled near-continuous acquisition of current–voltage relationships during solution exchange and temperature transitions. Thermal sensitivity of TMEM206-mediated currents was assessed using the automated perfusion system of the Patchliner. Briefly, 200 µL of extracellular solution was aspirated into the pipetting system, heated to the target temperature (20–45 °C), and then rapidly applied to the recorded cell by the robotic arm. Peak currents evoked at each temperature were extracted from the current–voltage relationships obtained during the voltage-ramp protocol. To account for cell-to-cell variability in channel expression, peak currents were normalized to the maximum current recorded within each individual cell across the tested temperature range. The ln of the normalized peak current (ln (I/I_max_)) was then plotted as a function of the reciprocal of absolute temperature (1000/T, K^−1^) to construct van’t Hoff plots. The linear region of each plot was fitted by least-squares regression, and the apparent activation enthalpy (ΔH) and entropy (ΔS) were extracted from the slope and intercept of the fit, respectively, according to:

$$ln(normalized\;I_{peak})\;=\;-\frac{\Delta H}{RT}\;+\frac{\;\Delta S}{R}$$

where R is the universal gas constant (1.987 cal·mol^−1^·K^−1^). Thermodynamic parameters are reported as mean ± SEM across 4–6 cells.

### 4.4. Fluorescence Spectroscopy

L-ANAP methyl ester was purchased from Asis Chem (Waltham, MA, USA). ANAP incorporation into TMEM206 was achieved by introducing an amber TAG codon at the desired position and co-transfecting cells with TMEM206 and pANAP plasmids. Five hours after transfection, ANAP dissolved in ethanol was added to the culture medium to a final concentration of 50 µM. After 2–3 days, cells were washed extensively with phosphate-buffered saline, replated onto 35-mm FluoroDishes (World Precision Instruments, Sarasota, FL, USA), and incubated overnight in ANAP-free medium before imaging. Epifluorescence imaging and spectroscopy were performed on an Olympus IX70 microscope (Olympus, Tokyo, Japan) equipped with a 40× oil-immersion objective (UPLANFLN, NA 1.4, Olympus, Tokyo, Japan). ANAP was excited using a 365 nm high-power LED (Prizmatix, Holon, Israel), with excitation light reflected by a T400lp dichroic mirror and fluorescence collected through a 435LP emission filter. Emission spectra were acquired using an Acton SpectraPro 2150i spectrograph (Princeton Instruments, Trenton, NJ, USA) equipped with a 150 grooves mm^−1^ grating and detected with an optiMOS CCD camera (QImaging, Tucson, AZ, USA). Images were acquired with 1 s exposure times. Emission spectra were collected over the 460–520 nm range.

To assess temperature-dependent spectral changes, emission spectra were recorded from individual cells at 20 °C and 37 °C under acidic conditions (pH 5.5). To assess proton-dependent spectral changes, spectra were recorded at 20 °C sequentially at pH 7.2 and pH 5.5. In all cases, spectra were acquired from regions of interest corresponding to the plasma membrane of individual transfected cells, identified by their ANAP fluorescence. Background fluorescence was estimated from adjacent non-transfected cells subjected to identical ANAP incubation conditions and subtracted prior to analysis. Emission spectra were normalized to their peak intensity to facilitate comparison of spectral positions across conditions. The peak emission wavelength was determined by fitting each spectrum to a bi-Gaussian function using OriginPro (OriginLab, Northampton, MA, USA). Temperature- and proton-dependent spectral shifts (Δλ) were calculated as the difference in fitted peak wavelength between conditions (Δλ_37–20°C_ and ΔλpH 5.5–pH7.2, respectively) and are reported as mean ± SEM across 7–15 cells. A threshold of |Δλ| ≥ 2 nm was applied to identify positions with significant spectral displacements.

### 4.5. Statistical Analysis of Spectral Shifts

Fluorescence changes were quantified using ImageJ (v1.51) and analyzed in OriginPro8.1. Normalized fluorescence vs. wavelength (nm) for each experiment was fitted to a Bi-Gaussian function:

(1) $$y=y_{0}+He^{-0.5{\left(\frac{x-x_{c}}{w_{1}}\right)}^{2}}(x<x_{c})$$

(2) $$y=y_{0}+He^{-0.5{\left(\frac{x-x_{c}}{w_{2}}\right)}^{2}}(x\geq x_{c})$$

where $y_{0}$ = Base, x_c_ = Center, H = Height, w_1_ = Width (left from x_c_) and w_2_ = Width (right from x_c_). Reported spectral shifts correspond to differences in the averaged fitted peak emission wavelength between experimental conditions ± standard error. To formally evaluate the significance of temperature-dependent spectral displacements at each ANAP-labeled position, Δλ values were computed as paired differences between fitted peak emission wavelengths at 37 °C and 20 °C for each individual cell. The distribution of Δλ at each position was characterized using nonparametric bootstrap resampling (10,000 iterations with replacement) to estimate 95% confidence intervals for the mean spectral shift. To test whether the mean Δλ at each position differed significantly from zero, one-sample permutation tests were performed by randomly reassigning the sign of each paired difference (10,000 permutations) and computing the proportion of permuted absolute mean values equal to or exceeding the observed absolute mean. The resulting *p*-values were corrected for multiple comparisons across the 10 labeled positions using the Benjamini–Hochberg false discovery rate (FDR) procedure, and adjusted q-values below 0.05 were considered statistically significant. Effect sizes were quantified using Cohen’s d, calculated as the ratio of the mean Δλ to the standard deviation across cells at each position. All analyses were performed in Python 3.12 using NumPy and SciPy libraries.

### 4.6. Structures Refinement

The experimental cryo-EM structure of TMEM206 captured in the resting state (PDB 7SQG [32]) was used as the starting model. Geometry minimization was performed in Phenix [41], and missing loops were further refined using the Prime module within the Schrödinger Maestro Suite (version 2022-04, Schrödinger, LLC, New York, NY, USA).

### 4.7. Molecular Dynamics Simulations

Molecular dynamics (MD) simulations were conducted using the Desmond engine (Schrödinger, LLC) with the OPLS4 force field for all protein and lipid interactions. The transmembrane protein was embedded into a pre-equilibrated 1-palmitoyl-2-oleoyl-sn-glycero-3-phosphocholine (POPC) bilayer and solvated using the SPC (Simple Point Charge) water model within an orthorhombic box, ensuring a minimum 10 Å buffer between the protein and box boundaries. The system was neutralized by adding 0.15 M NaCl to mimic physiological conditions. Following the default Desmond relaxation protocol, production runs were performed in the NPT ensemble at two distinct temperatures: 10 °C (283.15 K) and 50 °C (323.15 K). Temperature and pressure were maintained via the Nose-Hoover chain thermostat and the Martyna–Tobias–Klein barostat at 1.01325 bar, respectively. To ensure statistical robustness and sufficient sampling of the conformational landscape, two independent replicas were executed for each temperature condition, using different initial random velocities. Each simulation was carried out for a duration of 1 µs. Long-range electrostatic interactions were calculated using the Smooth Particle Mesh Ewald (PME) method with a 9.0 Å cut-off, and the RESPA integrator was employed with a time step of 2.0 fs for bonded interactions. Simulations were visualized and analyzed using VMD [42] and MD Analysis [43].

### 4.8. Dynamic Cross Correlation Analyses

To evaluate the effect of temperature on the protein dynamics, dynamical cross-correlation maps [44] were calculated using the Bio3D package in R 4.0 [45]. Dynamic cross-correlation matrices with coefficients were determined from the positions of the main chain Cα atoms in amino acids i and j with positions r_i_ and r_j_. Δr_i_ and Δr_j_ determine the displacement of the ith Cα from its mean position over the entire trajectory.

C_ij_ = ⟨Δr_i_·Δr_j_⟩/⟨Δr_i2_⟩⟨Δr_j2_⟩ (3)

## Data Availability

The original contributions presented in this study are included in the article. Further inquiries can be directed to the corresponding author.

## Supplementary Materials

The following supporting information can be downloaded at: https://www.mdpi.com/article/10.3390/ijms27114784/s1.
