# Supplementary material for: Conformational Fingerprints Underlying Thermal Modulation of PAC/TMEM206 Gating
Source: Int J Mol Sci. 2026 May 26;27(11):4784. doi: 10.3390/ijms27114784 (PMC13256793; doi:10.3390/ijms27114784)
Supplement: Supplementary file 1 [file ijms-27-04784-s001.zip › ijms-4289587-supplementary.pdf]

Supplementary files

Figure S1

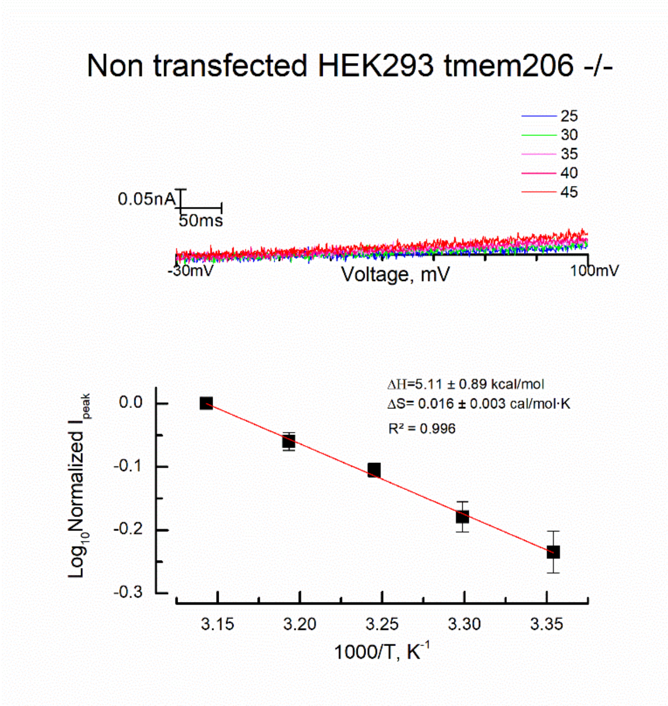

Figure S1. Temperature-dependent currents in non-transfected HEK293 tmem206⁻/⁻ cells confirm the absence of endogenous PAC-like activity. (Upper panel) Representative current traces recorded from non-transfected HEK293 tmem206⁻/⁻ cells in response to voltage ramps from −30 to +100 mV at temperatures ranging from 25°C to 45°C under acidic conditions (pH 5.5). Color code as indicated. Current amplitudes are negligible across the entire temperature range, confirming the absence of endogenous temperature-sensitive chloride currents in this cell line. (Lower panel) van't Hoff plot of the $\log_{10}$-normalized peak current versus 1000/T for non-transfected cells (n = 3). The linear fit yields ΔH = 5.11 ± 0.89 kcal/mol and ΔS = 0.016 ± 0.003 cal/mol·K (R^2 = 0.996), values substantially lower than those obtained for PAC-expressing cells, establishing the thermal background of the recording system.

Figure S2

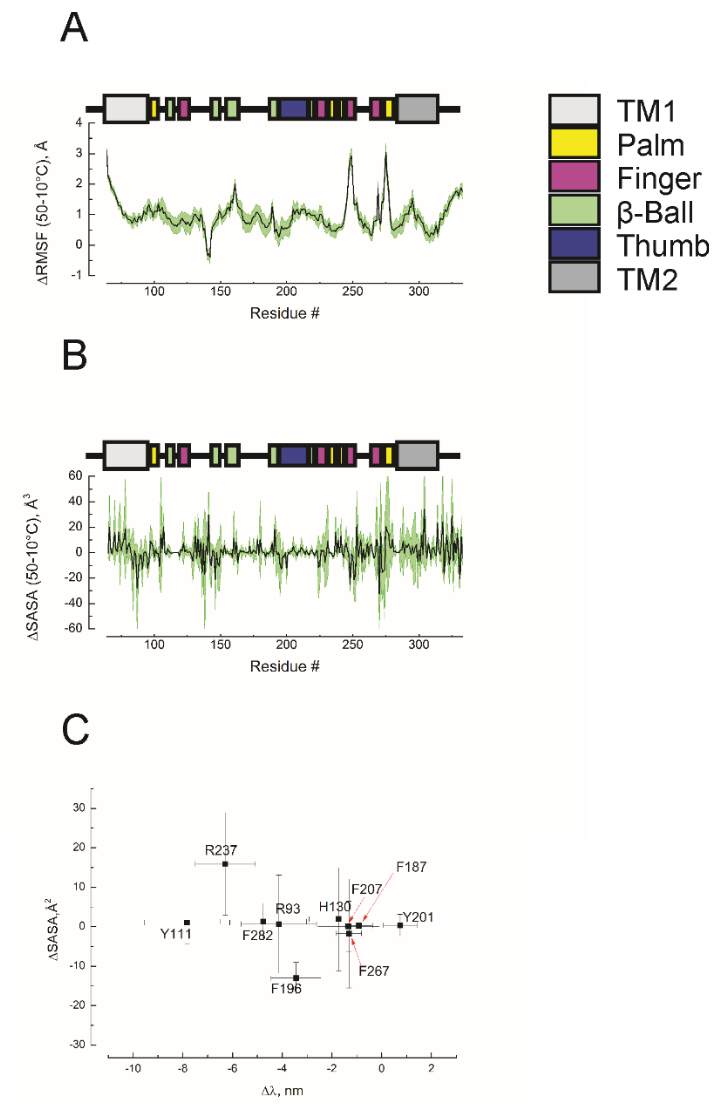

Figure S2. Temperature-dependent changes in PAC flexibility and solvent exposure from molecular dynamics simulations. (A) Per-residue difference in root mean square fluctuation (ΔRMSF, 50°C − 10°C) plotted as a function of residue number. The domain organization is shown above the plot. Black line indicates the mean across two independent replicas; green shading indicates the range between replicas. Elevated ΔRMSF values indicate residues that become more flexible upon heating, with the largest increases localized to the N-terminal portion of TM1, the Palm domain, and the C-terminal region of TM2. (B) Per-residue difference in solvent-accessible surface area (ΔSASA, 50°C − 10°C) as a function of residue number. Color code as in panel A. Temperature-dependent changes in solvent exposure are spatially restricted rather than globally distributed, involving selected portions of the extracellular region, the central β-sheet scaffold, subunit interfaces, and the lower pore-coupling region. (C) Scatter plot of the experimentally determined temperature-dependent spectral shift (Δλ, nm) versus the MD-derived ΔSASA (Å²) for each of the 10 ANAP-labeled positions. Error bars represent mean ± SEM.
